# Resilience in Japanese Older Immigrants in Canada and the Role of Community Support During the COVID-19 Pandemic

**DOI:** 10.1007/s10823-025-09526-z

**Published:** 2025-03-24

**Authors:** Mineko Wada, Sarah L. Canham

**Affiliations:** 1 Graduate School of Biomedical and Health Sciences, Hiroshima University, 1-2-3 Kasumi, Minami-Ku, Hiroshima, Japan; 2 College of Social Work, University of Utah, Salt Lake City, UT USA

**Keywords:** Aging in place, Social connection, Immigration, Service delivery, Asian American/Pacific Islander older adults

## Abstract

Japanese people make up a small proportion of the population in Canada, and there are limited resources to meet their specific needs. Thus, older Japanese Canadians were particularly affected by disrupted support and service systems when COVID-19 public health orders were implemented. The objective of this study was to explore how Japanese older immigrants cultivated resilience in overcoming challenges during the COVID-19 pandemic and how a community service agency supported the process. In this qualitative study, seven community-dwelling Japanese older immigrants and five agency staff participated in semi-structured interviews. Interviews were thematically analyzed using a conceptual lens of resilience, which refers to the ability to survive and thrive in the face of adverse life experiences. Our analysis yielded three themes: (1) Challenges and concerns associated with digital literacy, English literacy, COVID-19, and the future; (2) Individual sources of physical, mental, and social resilience; and (3) Agency-supported sources of resilience that enable management of health, safety, and daily life, connection, and belonging. The findings advance our understanding of the ways in which older immigrants cultivate resilience in the face of adversity and how programs and services can help older immigrants cope with adversity to meet their needs. Implications for service provision include ensuring systems are in place to digitally connect older adults to programs, support home maintenance and housekeeping, and engage older adults in the development of new programs.

Resilience has received increased attention in research that investigates how people experienced the pandemic and adapted to the ‘new normal,’ or new ways of life, and the research focusing on older adults is no exception. Resilience refers to the ability to cope with adverse situations (Bolton et al., 2016; Chen, 2020; Manning & Bouchard, 2020). The ecopsychosocial resilience framework developed by Windle and Bennett (2012) suggests that resilience is determined by the interaction between an individual and their environment (i.e., community and society). The conceptual model of resilience developed by Canham et al. (2020) delineates factors of resilience at the individual (micro), interpersonal (meso), socio-structural (exo), and socio-cultural (macro) levels and suggests that an individual’s resilience is determined by the dynamic interaction between multiple factors within each level. The individual factors include adaptive capacity, self-direction and determination, and sense of purpose; the interpersonal factors are primarily informal support networks (e.g., family, friends, neighbours); the socio-structural factors include formal health and social supports, community and public services, policies, and laws; and the socio-cultural factors include socio-cultural beliefs and perceptions (Canham et al., 2020).

Research suggests that, overall, older adults are not only resilient when confronted with adversity and hardship in later life (Wiles et al., 2019), but also perceive themselves as experienced, resourceful, and able to cope with adversity (Manning & Bouchard, 2020; Staudinger et al., 1993). Manning and Bouchard (2020) identified key components of resilience in later life, which include holding a resilient self (i.e., “a person’s ability to overcome adversity and hardship and the process of bouncing back” (p. 7); cultivating social support networks and resources; reframing hardship and adversity in a positive light; practicing gratitude; and perceiving hardships as simply a part of life rather than extraordinary.

Research has explored individual level facilitators of resilience for older adults during the COVID-19 pandemic, including staying busy (Amieva et al., 2021; Fuller & Huseth-Zosel, 2021), maintaining a positive mindset (Amieva et al., 2021; Fuller & Huseth-Zosel, 2021), having life purpose (Perez-Rojo et al., 2022), positively perceiving one’s aging and health (Losada-Baltar et al., 2021; Perez-Rojo et al., 2022), access to the outdoors and exercising regularly (Greer et al., 2023b; Hanna et al., 2022; Vannini et al., 2021), and adapting to the digital world (Amieva et al., 2021; Hanna et al., 2022; Lee et al., 2022). At an interpersonal level, facilitators include accessing social support (e.g., family, community organizations) (Fiocco et al., 2021; Fuller & Huseth-Zosel, 2021; Hanna et al., 2022; Park et al., 2021) and helping others (Vannini et al., 2021). Finally, socio-structural level facilitators of resilience include the availability of support networks (Fuller & Huseth-Zosel, 2021; Hanna et al., 2022; Lee et al., 2022) and information on available resources (Klasa et al., 2020, 2021; Lee et al., 2022).

Despite this extensive research, there has been little investigation into older immigrants’ experiences and resilience during the COVID-19 pandemic. While some studies suggest that immigrants’ experiences were similar to those of native-born individuals (e.g., anxiety, fear of infection, positive perspectives on lifestyle changes) (Leung et al., 2023), others suggest that immigrants appeared to experience the pandemic more negatively than their native-born counterparts. For example, Solà-Sales et al. (2021) found that in Spain immigrants experienced depression, anxiety, and stress more than non-immigrants during the pandemic. Amoako and MacEachen (2021) found that sub-Saharan African immigrants in Canada experienced racial harassment, discrimination, and stigma during the pandemic, which limited their access to health services and resources. And several studies showed that immigrants who were older, a member of a more visible minority group, and had low English literacy experienced distinct challenges during the pandemic (Bouka & Bouka, 2020; Mesa Vieira et al., 2020; Wang et al., 2021) due to language barriers and fewer social support networks compared to their native-born counterparts (Frank, 2020; Greer et al., 2023b).

The Japanese Canadian narrative began with the arrival of Manzo Nagano in 1877, who became the first documented Japanese immigrant to settle in Canada (National Association of Japanese Canadians, n.d.). In the years that followed, a significant wave of Japanese immigrants established themselves in the country, primarily engaging in agricultural and fishing pursuits. Little Tokyo—Canada’s pioneering Japanese neighbourhood—emerged in the 1880s in Vancouver’s Gastown district, where a Japanese sawmill workers’ initiative to house and mentor fellow countrymen in the lumber industry sparked the formation of a vibrant community (Ohki, 2024). The establishment of the Japanese Association of Canada in Vancouver in 1909 marked a significant milestone in community organization, while Little Tokyo served as the cultural and economic nucleus, providing essential services and fostering community cohesion (Ohki, 2024). During the Pacific War, approximately 21,000 Japanese Canadians (over 90%) were interned, and their citizenship was suspended, which fragmented once-thriving cultural institutions including religious organizations and Japanese language schools (Ohki, 2024). Since Japanese Canadians gained full privilege as Canadian citizens in 1977, the Japanese Canadian community has been expanding (Ohki, 2024).

In 2021, Japanese Canadians made up only 0.36% of the total population in Canada (Statistics Canada, 2023). The proportion of Japanese Canadians is small, while other East Asian ethno-cultural communities, such as Chinese Canadians and Korean Canadians account for 4.72% and 0.60% of Canada’s total population, respectively (Statistics Canada, 2023). Due to the small community, resources designed to meet Japanese Canadians’ distinct needs are limited and dispersed compared to resources available to the larger immigrant groups or non-immigrant populations. If the English literacy level of a Japanese Canadian is low, they have no choice but to rely on Japanese community, making them vulnerable. Thus, older Japanese Canadians were particularly affected by disrupted support and service systems when COVID-19 public health orders were implemented. Compared with other older adults, older Japanese Canadians generally have a higher risk of experiencing social isolation and difficulty accessing resources to help them meet their daily needs (National Seniors Council, 2017), especially if they do not communicate fluently in English (De Jong Gierveld et al., 2015), have limited technical knowledge and skills, or have restricted or no access to modern technologies that help them participate in social activities (Lu et al., 2023). Furthermore, public information is rarely translated into Japanese, exacerbating the vulnerability of older Japanese immigrants.

Research has shown that community organizations played a significant role in supporting older adults during the pandemic (Greer et al., 2023a), including outreach to older immigrants and refugees to solicit their needs and administer services—e.g., Spanish-speaking older immigrants in Florida (Calvo & Waters, 2023), older refugees from Afghanistan, the Middle East, and North Africa in California (Siddiq et al., 2023). Supports provided include informing in the first language of immigrants and refugees how to find food aid, preparing at-home meals and organizing and hosting activities to mitigate social isolation and sustain physical, mental, and emotional health (Calvo & Waters, 2023; Gallo & Wilber, 2021; Lee et al., 2022; Poulin et al., 2021; Siddiq et al., 2023). Additionally, some community organizations created online support groups for older adults while informing them about free resources via flyers and regularly checking in by phone (Lee et al., 2022).

While resilience has been investigated among general populations of older adults during the pandemic, little is known about immigrants’ resilience during this time. The objective of this study was to explore how Japanese older immigrants in Canada cultivated resilience in overcoming challenges during the COVID-19 pandemic and how one community service agency, Fuji Organization (pseudonym), supported the process.

## Methods

This is a qualitative study informed by Frechette et al.’s (2020) interpretive phenomenological inquiry, designed to explore the lived experiences of individuals by taking into consideration the social and cultural contexts in which they are situated. We conducted 12 semi-structured interviews. All data were thematically analyzed by drawing from Braun and Clarke’s (2006, 2012) techniques. Ethics approval was obtained from Adler University Research Ethics Board.

### Research Context

Fuji Organization, an association of Japanese community volunteers in Vancouver, British Columbia, Canada, provides services and programs to Japanese Canadian older adults and other Japanese Canadian community groups and celebrates Japanese culture. Guided by the foundational principles of active community engagement, support for healthy living, and strategic inter-organizational collaborations, Fuji Organization provides direct services that help older immigrants with housing, medical interpretation, transportation to medical appointments, and paperwork for government, as well as programs for social connections, and recreational and traditional Japanese cultural activities. Following the onset of the COVID-19 outbreak, Fuji Organization consistently supported its older Japanese Canadian members through phone calls and communication via other technologies.

### Participants

We recruited older community-dwelling Japanese immigrants and Fuji Organization staff members for this study. All older adults aged 65 years or older and members of Fuji Organization and all Fuji Organization staff members (regardless of age) were eligible to participate. Purposive and snowball sampling were used to recruit participants. To recruit older adults, recruitment flyers outlining study details (e.g., objectives, eligibility criteria, data collection protocol) were posted on Fuji Organization’s website and announced via the programs and services Fuji Organization offered. To recruit Fuji Organization staff, emails were sent to inform staff about the study. Additionally, at the end of each interview, participants were invited to circulate recruitment information to other potential participants.

Seven community-dwelling older adults and five staff (Table 1) provided informed consent to participate in a semi-structured interview. Four older adults were women and three were men; all were in their 70s; and had resided in Canada for approximately 10–55 years. Three of the Fuji Organization staff participants were women and two were men. All participants were assigned a pseudonym to ensure confidentiality.

**Table 1:** Participant characteristics

Pseudonym		Age	Gender	Length of residence in Canada
Older adults				
	Megu	75–79	Woman	Approx. 50 years
	Midori	70’s	Woman	Approx. 45 years
	Teresa	70’s	Woman	Approx. 25 years
	Yuzo	70–74	Man	Approx. 10 years
	Ken	75–79	Man	Approx. 55 years
	Akira	70–74	Man	Approx. 35 years
	Ivone	70’s	Woman	Approx. 50 years
Staff				
	Yoshiko		Woman	
	Tom		Man	
	Lucas		Man	
	Kai		Woman	
	Leo		Woman	

### Data Collection

We conducted semi-structured, one-on-one, in-depth interviews as this method allowed us to elicit descriptions of complex human experiences and the meanings that individuals attributed to those experiences (Hesse-Biber, 2016). We interviewed each participant once via Zoom or phone between March and June 2021. All interviews were conducted in Japanese except one in English. Interviews lasted approximately 60–110 min (average: 84 min for older adults and 58 min for staff) and were digitally recorded with participants’ written consent. The aim of the interviews with older Japanese Canadians was to elicit (1) how they accessed information about COVID-19; (2) how they met their daily needs during the pandemic; and (3) what services and supports could have helped them better cope with challenges and manage their daily activities. The interviews with Fuji Organization staff aimed to understand (1) how they perceived the challenges that older Japanese Canadians faced in meeting their daily needs during the pandemic and accessing information about COVID-19; (2) how Fuji Organization did or did not adapt their services and programs to support older Japanese Canadians; and (3) what services, programs, and systems needed to be developed to better meet the needs of older Japanese Canadians.

### Data Analysis

The digitally recorded interviews were transcribed verbatim, de-identified to ensure confidentiality, translated into English, and thematically analyzed guided by Braun and Clarke’s(2006, 2012) approach. The first author initiated analysis by exhaustively reading the transcripts and then inductively developing an initial set of descriptive codes using the qualitative data analysis software NVivo 12, which supported data organization and management. Initial codes were amalgamated into categories that captured the semantic content of the data and then collated into higher-level categories relevant to the overarching themes that encapsulated (1) older Japanese Canadians’ experiences of the COVID-19 pandemic, (2) staff perceptions of the challenges faced by older Japanese Canadians, and (3) changes to services and programs provided by Fuji Organization during the pandemic. The authors discussed the categories, themes, and interpretation of the data, which enabled us to understand the data in a more nuanced manner and to reach consensus on the themes developed.

## Findings

Thematic analysis yielded three themes: (1) Challenges and concerns associated with digital and English literacy, COVID-19, and the future; (2) Individual sources of physical, mental, and social resilience; and (3) Agency-supported sources of resilience that enable management of health, safety, and daily life, connection, and belonging.

### Challenges and Concerns

The challenges experienced by Japanese older immigrants included hardships associated with digital literacy, English literacy, fear of COVID-19 infection, and concerns about the future.

#### Limited Digital and English Literacy

Older adult participants recounted negative experiences using technology for communicating with friends and families and getting information. For example, Ivonne, who regularly used computers for her personal and business needs before the pandemic, expressed frustration that she could not contact service agencies by phone if she could not find information online. Another older adult, Akira, noted, “It’s a challenge for me to use computers and email and take online programs because I am analog.” For older adults like Akira, limited digital literacy was a barrier to accessing information online.

There was also a discussion about the drawbacks of overusing communication technologies. Midori acknowledged that she was grateful to be able to participate in online social activities and programs, but she also noted:

I started to feel like a Zoomer or YouTuber. Using it too much doesn’t feel good. I need to control the time I spend online because I want and appreciate time to be by myself. I began to feel time was racing by a bit because I was spending too much time online.… I need to re-evaluate and reorganize my time for online and offline activities.

Limited English literacy was another challenge Japanese older immigrants reported experiencing during the pandemic that impacted their ability to manage their daily lives, use medical services, and access COVID-19-related information. Yoshiko, a Fuji Organization staff member, highlighted that the COVID-19 public health orders disrupted older Japanese Canadians’ support networks, which hindered their opportunities for support with their everyday lives. She explained:

Most of the older adults we visit have difficulty communicating in English. Even if they don’t have problems with English at a younger age, it gets harder as they get older; they’d gradually start losing their second language. And if they don’t have family around, it would be hard for them to ask for help. Unfortunately, the pandemic made their situation worse. They have no one to ask for help because most activities have been cancelled. No opportunities to gather, no one to ask for help.

Older adult participants reported that they relied heavily on Fuji Organization when accessing and trying to understand information about COVID-19 and public health measures. For example, Ken acknowledged:

I couldn’t have been vaccinated for COVID-19 had I not received a newsletter from Fuji Organization informing me about the vaccination.…English? I’m not good at it at all.…So I couldn’t understand information about the vaccination if it was written in English, especially because it includes medical terms and jargons. I’m grateful that I’ve been vaccinated and feel secure.

Limited English literacy significantly impacted Japanese older immigrants who wanted to access healthcare services during the pandemic as many healthcare services integrated e-health into their practice (e.g., using email, phone calls, and smartphone apps). As the digitization of healthcare services minimized in-person contact between healthcare providers and all patients, it further challenged older Japanese Canadians to communicate and explain their symptoms and health issues. Midori explained:

We had to communicate with healthcare professionals via email or phone.…My husband was told to take examinations by his physician…and then he received the results by email. Considering the way he had to communicate with the physician and interpret the exam results, I think many older Japanese Canadians face a lot of challenges if their family doctors don’t speak Japanese.…Some of the Japanese immigrants that I know of don’t seem to understand much of what their family doctors said.

#### Fear of COVID-19 Infection

Older adult participants recounted how frightened they were of contracting COVID-19 when provincial public health orders restricted non-essential outings. Megu was particularly frightened during the initial phase of the pandemic because they “didn’t know anything [about] the coronavirus…like, how contagious it is.” Teresa noted that stories she heard from people, combined with media reports on the pandemic, fed her growing fear that she might be infected if she took public transit or walked by people on the street. She said:

I was afraid if I took this train, I might be infected by COVID. If I passed by people on the street, there might be a risk of getting COVID.…I became so worried that I was fearful to even go out.

#### Concern About the Future

Finally, older adults revealed that their concerns about their future life were exacerbated during the pandemic. For example, Teresa said:

We’ve been just myself and my husband with no children. I’m particularly concerned about our future because of that.…as we got into our 70s, we started to experience more challenges in our daily lives, and our health became more of a concern.…I wonder if we can still live at home independently if we have health issues.

Teresa explained how her friends were supported by their children in their everyday lives during the pandemic (e.g., with grocery shopping, transportation, and emotional support). Her concerns about her future, particularly if her husband’s or her health were to deteriorate, increased because of her limited social networks and options for immediate assistance.

### Individual Sources of Physical, Mental, and Social Resilience

In response to the challenges presented by the COVID-19 pandemic, Japanese older immigrants stayed active by reinitiating or sustaining regular exercise habits, nurturing and sustaining a positive mindset, and maintaining social connections—all components of resilience.

Older adult participants reported that they continued exercising regularly to maintain their health during the pandemic. Akira, an older adult who has diabetes, noted that he kept up his walking habit: “Every day I take a walk for more than three hours, 24,000 to 30,000 steps in total… I’ve been doing that for years because I have diabetes.…I’m responsible for my own health.” Another older adult, Teresa, described re-establishing their exercise habits during the pandemic:

I walked around my house in sunny afternoons. There were few people in this neighbourhood. I enjoyed looking around, trees, cherry blossoms, and so on.…Inside the house, whenever I learn a new exercise, like squats, from Fuji Organization’s online program, I integrate it into my exercise program, which now takes 30 min.… I do it twice a day, in the morning and the evening.

While some participants struggled with adapting to sudden, unprecedented changes (e.g., cancellations of social events) arising from pandemic-related public health measures, others recounted that they stayed positive and found alternative ways to fill their calendars. Midori described her experience in the initial phase of the pandemic:

Before the pandemic, my calendar was full of socials and volunteer activities.…all of [a] sudden everything was cancelled.…Initially I wondered what I was gonna do, but I took the situation positively and decided to use the time to clear clutter at home, which I had wanted to do for a long time.…When I’d done it, I felt good and refreshed.…I would have felt anxious if I had been sitting doing nothing. So I found other ways to stay busy and did what I could at that time, like decluttering and doing crafts.

Similarly, reflecting on the past year, Teresa acknowledged that technology helped her find the information she needed and to cope with the challenges she faced. She said:

Thanks to the online and phone programs [Fuji Organization] launched quickly, we could start connecting again.…I decided to participate in the programs no matter what. I was fully committed.…Through participating in them, I learned I could not only connect with my friends but also find information by using technology.…I learned [that] we can overcome challenges if we actively find a way to address them instead of doing nothing and just complaining.

While older men study participants noted that they enjoyed spending time alone or primarily with their family, older women reported on ways they sustained social connections outside the home amidst physical distancing precautions. For example, Midori said, “I continue to contact my friends regularly during the pandemic, which helps uplift my feelings, especially when I talk to one of them who is extremely positive.” Megu actively engaged in social activities:

There is a community garden near my apartment, and I am a member of it. It is really fun to garden in a group. Before the pandemic, we grew and harvested vegetables. We then cooked them at home and had a potluck lunch. We used to be very active.…now…we are working in a small group– like 4–5 members at a time.…but I still love seeing them and chatting with them. It really helped me go through difficult time last summer.

Like Midori and Megu, other female older adult participants stayed socially connected, which might have mitigated feelings of loneliness and isolation.

### Agency-Supported Sources of Resilience

Fuji Organization developed and delivered a variety of new services and programs to meet the needs for management of health, safety, and daily life, social connection, and belonging for their older Japanese members.

#### Redesigning Services To Support Older Adults’ Health, Safety, and Daily Life

As soon as the pandemic started, Fuji Organization recognized that helping older Japanese Canadians meet their day-to-day needs was critical and should be their priority. Leo, a staff member, reflected on how Fuji Organization redesigned their services and programs to align with this priority:

Before the pandemic, we focused primarily on organizing and providing recreations and activities…. Since the pandemic began, our focus has been to help older Japanese Canadians stay safe and manage their daily lives. For example, we’ve been distributing information on the coronavirus, disease control measures put in place, and vaccination. And some older adults asked us for help as they felt mentally drained and felt stuck at home because they were afraid of going out and had no family around, and so we started helping meet individual needs one by one.

To meet individual needs, Fuji Organization started a program to help older adults with light housekeeping and maintenance. Yoshiko explained, “We are offering home visit services. In the initial pandemic year, I supported 50 older adults who were frail and had no family.…I visited some of them to help [with] anything they needed, like grocery shopping.” Another staff member, Tom, pointed out the benefits of the home visits:

I just went the other day to help a 94-year-old lady who lives alone in her apartment. She has a fall-prevention pendant, but the battery ran out… We were able to replace the battery very simply. Otherwise, she would have had to call in a professional maintenance person to do that. It would have taken time and also some additional funding.

Fuji Organization also more than doubled their lunch delivery services to not only serve nutritious meals, but to help older adults mitigate feelings of isolation and loneliness. Tom recounted:

We’re finding that a lot of people are feeling very lonely, being isolated, and couldn’t go out to Japanese restaurants even if there are [any] in their locality.…We are expanding [the lunch delivery program] not only to be in Vancouver but also to be in [Metro Vancouver Area]. So this has been a significant ramp-up in terms of the number of lunches that we deliver and the number of volunteers that we’ve had to mobilize, which is very difficult in the midst of COVID.…But it’s been extremely gratifying, I think people are really, enjoyed it and appreciate it that I’ve listened to many people talk about it… our lunches are different in that, they are different in each week. They’re sort of like home-made…very, very attractive nutritious and tasty lunches.

Tom acknowledged the importance of addressing older Japanese Canadians’ feelings of loneliness and isolation by making lunches “home-made,” culturally familiar, and “different each week.” Another staff member, Leo, noted that Fuji Organization included its newsletters and local Japanese magazines in the lunch bags to help older Japanese Canadians stay informed about COVID-19, public health measures, and other local news, all of which helped older Japanese Canadians sustain their health and safety.

#### Creating and Sustaining Digital Social Spaces

Fuji Organization staff members reported that when Fuji Organization cancelled all in-person activities and programs in the initial stage of the pandemic, they were concerned about the impact of the cancellations on older Japanese Canadians’ mental and social health, particularly if they lived alone and had no family nearby. Therefore, Fuji Organization quickly launched virtual programs to help their members engage socially. Tom explained:

With the pandemic, all of our in-person activities such as the lunches at our facility…exercise programs…had to be closed down. Also,…we had to cancel our friendly visitation program, where people actually went out to meet with others. So this was quite a significant blow, so how we were trying to compensate for that was it pivoted as much as possible to either telephone or virtual, such as Zoom-type activities.…then as it became clear that this was going to continue for quite a bit longer…we started to offer what we called Zoom Lounge…where seniors could login via Zoom to have chats and to meet with each other and to speak also to our staff members.

Leo emphasized that Fuji Organization prioritized creating and sustaining digital social spaces, like Zoom, particularly for people who lived alone so they could interact with others in Japanese.

While developing online programs for older Japanese Canadians, Fuji Organization staff identified their priority of helping older Japanese Canadians learn communication technologies and putting a support system in place. Tom explained that Fuji Organization used multiple methods, including providing “detailed instructions” via hard copy, phone, and in-person consultations to help older adults set up Wi-Fi and Zoom. Additionally, Fuji Organization provided offline check-ins with older adults by assigning volunteers to interact with older adults when delivering lunch. Lucas, who volunteered for lunch delivery services, reported:

I am always in charge of delivering a lunch box to five older Japanese Canadians.…I try to have a conversation with each of them.…Fuji Organization requested that when delivering lunch, volunteers check in with older adults and report back if they don’t look well or if they show any signs of—for example, health deterioration or trouble that alert to the volunteers.

These informal check-ins were appreciated by the older adults. Kai, another volunteer, noted that those who resided in long-term care homes looked forward to not only the lunch boxes from Fuji Organization, but also the chance to chat with volunteers in Japanese because there were very few or no people with whom they could converse with in Japanese.

#### Integrating Culture into Program Considerations

Fuji Organization staff highlighted that they tried to integrate Japanese culture into their services and programs to foster a sense of belonging. For example, Tom noted:

On Kodomo-no-hi (Children’s Day), we had a special card for everybody and on Girls’ Day, we also had a Hina Ningyo (doll), little origami… We’re trying to keep each different holiday remembered as well.…For seniors, it even seems more isolating.

During the pandemic, Fuji Organization gradually resumed programs associated with Japanese culture online or by telephone, including telling nursery rhymes and Rakugo (traditional Japanese story-telling entertainment).

To meet the needs of all older adults, Tom highlighted how critical it is be conscious of and consider Japanese older men’s values and characteristics when developing programs and services. He noted:

We find that a lot of Japanese Canadian males tend to be not too open to doing different things,…because…they don’t come forward to complain or to seek help, there is a bigger need for us to go out and find them.…It’s important for us to be able to reach out and assure people that…we do care about them, that their needs are being considered and that we’re trying to be of help when they need it.

Considering Japanese men’s hesitance to ask for help, Tom suggested that Fuji Organization reach out to men in the community who might be hiding their challenges.

## Discussion

This paper explored challenges that older Japanese Canadians experienced during the COVID-19 pandemic, how they cultivated resilience in overcoming challenges, and how one community service agency supported processes of resilience. Our findings revealed that the older Japanese Canadians experienced challenges related to limited digital and English literacy, fear of COVID-19 infection, and concerns about the future. However, resilience was cultivated by reinitiating or sustaining exercise habits, nurturing and sustaining a positive mindset, and maintaining social connections. The Fuji Organization supported older Japanese Canadians’ resilience by developing services and programs designed to help them manage their health, safety, and daily lives, as well as socially connect and maintain feelings of belonging to the Japanese community through cultural considerations.

Our findings of the challenges and concerns experienced by Japanese older immigrants during the pandemic echo evidence from previous research with non-immigrant populations of older adults. For example, our participants identified distress resulting from a lack of digital competence, fear of COVID-19, and anxiety about their health and ability to age in place, just as older adults have reported in previous studies (Amieva et al., 2021; Hanna et al., 2022; Igarashi et al., 2022; Islam & Akter, 2021; Lee et al., 2022; Vannini et al., 2021). Additionally, challenges managing activities of daily living (e.g., changing batteries) and disrupted routines and meaningful activities have similarly been identified in prior research (Igarashi et al., 2022; Jawaid, 2020). While previous research highlights Chinese immigrants’ mental health challenges that were associated with perceived or experienced racism and discrimination (Gao, 2021; Yu et al., 2022), the Japanese older immigrants in this study did not report this as a challenge.

Our participants highlighted that though they had lived in Canada for decades, a low level of English literacy was a predominant, fundamental challenge that led to difficulties managing daily life, using medical services, and accessing critical information about COVID-19 and other matters. Previous research indicates that immigration status, often associated with language competency, is a social determinant of one’s resilience (Canham et al., 2020), and that English proficiency among immigrants is critical to health literacy (Jacobson et al., 2016; Todd & Hoffman-Goetz, 2011), social integration (Heizmann & Böhnke, 2016), and economic advancement (Heizmann & Böhnke, 2016; Rivera-Batiz, 1990). Thus, a low level of English literacy makes it difficult for older immigrants to manage multiple areas of their daily lives. Further, the trajectory of English literacy among older immigrants may be potentially compromised by cognitive decline as extant research indicates that bilingual individuals experiencing dementia demonstrate diminished linguistic functionality in their secondary language relative to their first or dominant language (Lind et al., 2018). However, the empirical research remains nuanced, with existing studies staying inconclusive on the complex interplay between bilingualism, cognitive decline, and language performance (Manchon et al., 2015; Mendez, 2019; Nanchen et al., 2017).

The public health measures that restricted social gatherings and non-essential outings limited older adult participants’ access to formal and informal support network resources. As the resources were already scarce because Japanese immigrants make up such a small segment of Canada’s population, the limited accessibility to the resources further challenged the ability of older adults to navigate the healthcare system, manage daily activities, and exchange and gather information in Japanese. Accessing support network resources allows older adults to communicate in their native language as they wish to verify information they learned in English or ask for help with managing their daily lives and health. The disruption to these resources resulted in lost opportunities to seek help and access updated COVID-related information, which can be difficult to understand because of jargon and technical terms.

Our findings contribute to knowledge on the ways in which older immigrants cultivate resilience in the face of adversity. The Japanese older adult participants in this study coped with pandemic-related challenges by staying physically, mentally, and socially active. Staying busy and positive, regularly exercising, and connecting socially have been previously identified as strategies for resilience (Amieva et al., 2021; Fuller & Huseth-Zosel, 2021; Hanna et al., 2022). In the initial phase of the pandemic, some older participants were dedicated to keeping or re-establishing regular exercise and social interaction, which was part of their adaptation to a disrupted daily life. According to the resilience model developed by Richardson (2002), an individual has opportunities to make decisions about how to respond to adversity in the process of resilience. The choice to sustain regular exercise and social connection was a way for older participants to maintain biopsychospiritual homeostasis. Keeping a part of routine established before the pandemic, whether physical or social, was critical to anchoring participants’ disrupted lives and maintain participants’ self-direction and determination—the individual factors of resilience (Canham et al., 2020).

Additionally, older participants reported having managed sudden life changes by reframing the adversity in a positive light, seeing it as an opportunity to do things they would not otherwise have done (e.g., clearing clutter). Manning and Bouchard (2020) have suggested that this reframing of challenges is one of the key components of resilience. Our analysis highlighted that some older adults sustained the agentic self of being resilient—“a resilient self” (Manning & Bouchard, 2020, p. 7)—and committed to mastering the use of technology and engaging in online programs, which strengthened their sense of a resilient self.

Study findings suggest that initiating or maintaining support networks that meet individual needs is critical to facilitating older Japanese Canadians’ resilience. First, the Fuji Organization expeditiously implemented digital intervention programs, specifically tailored online and telephone platforms designed to facilitate older Japanese Canadians’ integration into digital sociocultural spaces. These strategic initiatives enabled older Japanese Canadians to reestablish meaningful connections with their ethnocultural community and simultaneously provided access to traditional Japanese cultural activities, such as nursery rhymes, music, haiku, and rakugo. Given the small size of the Japanese Canadian community, cultivating these digital socio-cultural spaces for older Japanese Canadians was crucial to generating opportunities for a sense of purpose—an important individual factor for resilience (Canham et al., 2020). The Fuji Organization also provided older adults with services and programs in Japanese. For example, to help older adults manage their health and safety, Fuji Organization kept members informed about COVID-19 and related public health measures by providing communications in Japanese, which nurtured older adults’ ability to adapt to life during the pandemic. Accessing information that helps individuals cope with hardship and adversity is key to resilience (Klasa et al., 2020, 2021; Lee et al., 2022), and Fuji Organization played a critical role in translating and distributing relevant information. Further, based on the communication with older adults in the initial phase of the pandemic, Fuji Organization reprioritized their programmatic foci and redeveloped services and programs to meet older adults’ needs for managing their health, safety, and daily lives; adapting to digitization and social reconnection; and sustaining a sense of belonging to Japanese culture. Transforming services and programs, Fuji Organization helped navigate older adults’ reintegration process.

Our findings have implications for how to shape future and existing programs and services to help older Japanese Canadians and other older immigrants meet their needs and cope with adversity. First, it is critical for community organizations to develop a support system to help older immigrants learn how to set up Wi-Fi, navigate the Internet, and use information and communication technologies (ICTs). Community organizations should consider delegating a staff member to supporting ICT use among older adults, including consultation and troubleshooting. Second, older immigrants may benefit from exploring, discussing, and co-developing various programs that could provide a balance between in-person and online activities as high Internet usage can inadvertently create a habit of excessive usage, which may affect a sense of control (Bertocchi et al., 2022; Busch et al., 2021). Third, it is essential that community organizations sustain and expand programs to help older immigrants with light housekeeping and house maintenance to support independent living in communities of choice. Finally, it is vital for community organizations to be conscious of and take into consideration older immigrants’ cultural values, beliefs, and characteristics when developing and providing services and programs. For example, community organizations might consider reaching out to older immigrants who hesitate to seek help.

This study is potentially limited in that it was conducted with participants from a single ethnic group of immigrants in one metropolitan area. Despite this, however, the in-depth data offers rich insight into the lived experiences of adversity and resilience among an understudied ethnic group. It is recommended that future research expand upon this study by conducting interviews with a larger sample of older Japanese immigrants and comparing data to older Japanese non-immigrants. Future research should also examine the avenues to resilience for other older immigrant groups who may not have many support options from culturally tailored organizations. Finally, while we have suggested the need to expand programs and services to help Japanese older immigrants better meet their needs and cope with adversity, future research should explore how Japanese older immigrants who are not supported through community service agencies cultivate resilience in the face of adversity.

## Conclusions

This paper explored how older Japanese Canadians cultivated resilience in overcoming challenges during the COVID-19 pandemic and how one community service agency supported older adults’ resilience. For Japanese older immigrants, low levels of English literacy can be a predominant challenge, yielding difficulties with management of daily life, using medical services, and accessing critical health information. While implementing support networks that meet individual needs is critical to promote Japanese older immigrants’ resilience at the socio-structural level, staying physically, mentally, and socially active was key to overcoming adversity at the individual and interpersonal levels.

## Data Availability

The data are not available due to data use agreement limitations.
